# The Role of Staging Laparoscopy for Gastric Cancer Patients: Current Evidence and Future Perspectives

**DOI:** 10.3390/cancers15133425

**Published:** 2023-06-30

**Authors:** Carlo Alberto Schena, Vito Laterza, Davide De Sio, Giuseppe Quero, Claudio Fiorillo, Gayani Gunawardena, Antonia Strippoli, Vincenzo Tondolo, Nicola de’Angelis, Sergio Alfieri, Fausto Rosa

**Affiliations:** 1 Digestive Surgery Unit, Fondazione Policlinico Universitario Agostino Gemelli IRCCS, 00168 Rome, Italy; 2 Unit of Colorectal and Digestive Surgery, DIGEST Department, Beaujon University Hospital, AP-HP, University of Paris Cité, Clichy, 92110 Paris, France; 3 Department of Digestive Surgery, Università Cattolica del Sacro Cuore, 00168 Rome, Italy; 4 Medical Oncology, Fondazione Policlinico Universitario Agostino Gemelli IRCCS, 00168 Rome, Italy; 5 Emergency and Trauma Surgery Unit, Fondazione Policlinico Universitario Agostino Gemelli IRCCS, 00168 Rome, Italy

**Keywords:** staging laparoscopy, advanced gastric cancer, gastric cancer staging, peritoneal carcinomatosis, peritoneal cytology, artificial intelligence

## Abstract

**Simple Summary:**

The presence of peritoneal carcinomatosis in patients with advanced gastric cancer has important implications for the management of this disease. For this reason, staging laparoscopy plays an important role in the preoperative diagnosis of peritoneal dissemination in these patients. A review of the current literature was performed in order to summarize the indications, advantages, and future perspectives of staging laparoscopy and peritoneal cytology in the diagnosis and management of advanced gastric cancer.

**Abstract:**

A significant proportion of patients diagnosed with gastric cancer is discovered with peritoneal metastases at laparotomy. Despite the continuous improvement in the performance of radiological imaging, the preoperative recognition of such an advanced disease is still challenging during the diagnostic work-up, since the sensitivity of CT scans to peritoneal carcinomatosis is not always adequate. Staging laparoscopy offers the chance to significantly increase the rate of promptly diagnosed peritoneal metastases, thus reducing the number of unnecessary laparotomies and modifying the initial treatment strategy of gastric cancer. The aim of this review was to provide a comprehensive summary of the current literature regarding the role of staging laparoscopy in the management of gastric cancer. Indications, techniques, accuracy, advantages, and limitations of staging laparoscopy and peritoneal cytology were discussed. Furthermore, a focus on current evidence regarding the application of artificial intelligence and image-guided surgery in staging laparoscopy was included in order to provide a picture of the future perspectives of this technique and its integration with modern tools in the preoperative management of gastric cancer.

## 1. Introduction

Staging laparoscopy (SL) is a minimally invasive diagnostic procedure used for gastrointestinal and gynecological tumor staging, including advanced gastric cancer (GC). During SL, a thorough exploration of the abdominal cavity is carried out to detect possible occult peritoneal metastases that might have been missed by preoperative imaging.

SL plays a key role in the preoperative assessment of GC, as the detection of peritoneal carcinomatosis is considered as distant metastasis by the 8th International Union for Cancer Control/American Joint Committee on Cancer TNM classification, ranking the disease as stage IV [1,2] and shifting the initial therapeutic management from upfront surgery toward systemic treatment.

Notwithstanding this, the importance of SL has long been debated since 1984. 

On the one hand, Shandall et al. questioned the value of laparoscopy, given the need for at least palliative surgery in a high percentage of patients [3]. Conversely, other authors, such as Gross et al., already recognized its role in identifying metastatic disease while carrying negligible morbidity, and thus avoiding unnecessary laparotomies [4,5]. 

Nowadays, the employment of new chemotherapy regimens, the technological improvement in diagnostics and surgery, and the multi-disciplinary decision making have expanded the boundaries of advanced GC treatment, paving the way to patient-tailored strategies and improving overall and disease-free survival [6,7,8,9]. 

However, despite computed tomography (CT) currently representing the gold standard technique for preoperative GC staging, its performance in detecting peritoneal dissemination is suboptimal and often relies on many indirect signs of carcinomatosis, such as ascites and omental retraction [10]. 

SL can provide supplementary information regarding GC peritoneal dissemination, also through peritoneal washing and cytological analysis [11]. 

For this reason, SL is recommended for preoperative staging of GC by several international guidelines, including those published by the National Comprehensive Cancer Network (NCCN) [12], European Society for Medical Oncology (ESMO) [13,14], Society of American Gastrointestinal and Endoscopic Surgeons (SAGES) [15], and the Japanese and Italian guidelines [16].

This review aims to provide an overview of SL for GC, its indications, benefits, limitations, and future perspectives.

A literature search was performed by screening MEDLINE, Cochrane Library, and EMBASE from inception to 1st April 2023. Specific research equations were designed for each database, using the following search equation: ((staging laparoscopy) OR (diagnostic laparoscopy)) AND (gastric cancer). After a first screening based on titles and abstracts, thirty-seven articles were selected and underwent full-text analysis. Relevant articles were reviewed, and their references were also screened for additional relevant studies. Only English-language articles were included.

## 2. Indications for Staging Laparoscopy (SL)

The most important aim of SL is to detect occult peritoneal disease through direct intraperitoneal view or cytological analysis after peritoneal washing, which cannot be preoperatively diagnosed by conventional radiological imaging [17].

The most important indications for SL according to National and International Guidelines are summarized in Table 1.

In Western countries, where the ratio of advanced–early GC is more common, there is a general indication for SL in the case of a resectable GC without distant metastases. In particular, according to the National Comprehensive Cancer Network (NCCN) Clinical Practice Guidelines in Oncology for GC [12], SL may be employed for the detection of radiographically occult metastases in patients considered for surgical resection without preoperative therapy when T3 and/or N+ GC has been diagnosed at preoperative imaging. Moreover, the panel recommends SL and cytology of peritoneal washings in medically fit patients with potentially resectable stage cT1b or higher locoregional disease when surgery and/or preoperative chemoradiation are considered. Finally, SL with cytology can be considered for medically fit patients with unresectable diseases [19].

As regards European Society for Medical Oncology (ESMO) guidelines [14], SL with peritoneal washings for malignant cells is recommended in all potentially resectable stage IB-III GC to exclude occult peritoneal metastases disease for patients who are also candidates for perioperative chemotherapy [III, B]. The panel recommends classifying peritoneal carcinomatosis according to the Peritoneal Carcinomatosis Index (PCI) [20,21].

According to the Society of American Gastrointestinal and Endoscopic Surgeons (SAGES) guidelines [15], SL is indicated for patients with cT3–4 N0 M0 GC. Additionally, the authors also suggested several contraindications, as follows: obstructive, hemorrhagic, or perforated GC needing palliative surgery; early-stage GC (T1–2); patient history of abdominal surgery due to the presence of adhesions that may preclude the procedure.

In Eastern countries, SL is carried out based on the results of two clinical trials of the Japan Clinical Oncology Group (JCOG), in which SL was systematically performed to confirm the eligibility criteria [22,23]. According to the 6th edition of the Japanese Gastric Cancer Treatment Guidelines [16], SL is weakly recommended to establish the therapeutic strategy for patients with advanced GC with a high risk of peritoneal carcinomatosis. Several studies investigating clinical, radiological, and pathological features associated with peritoneal carcinomatosis or cytology identified linitis plastica, Bormann type 3–4, poor histological poor differentiation, and suspected lymphadenopathy at CT scan as risk factors at multivariate analysis [24,25,26,27,28,29].

## 3. Technique of Staging Laparoscopy with Peritoneal Cytology (PC)

**Figure 1:**
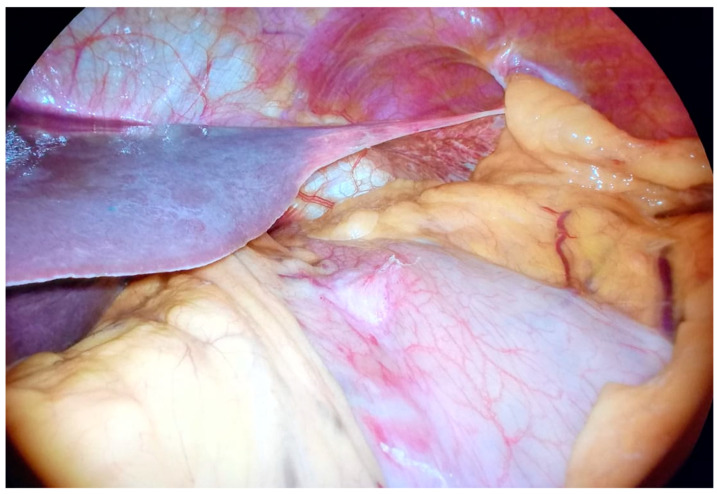
Gastric cancer with serosal invasion.

**Figure 2:**
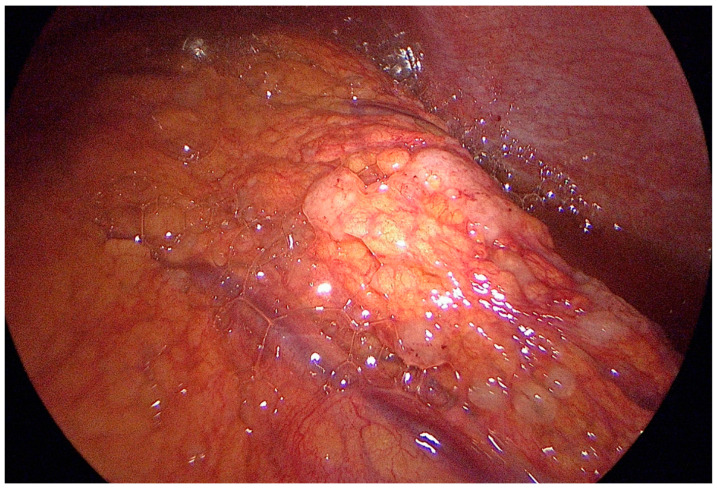
Peritoneal carcinomatosis of the greater omentum.

**Figure 3:**
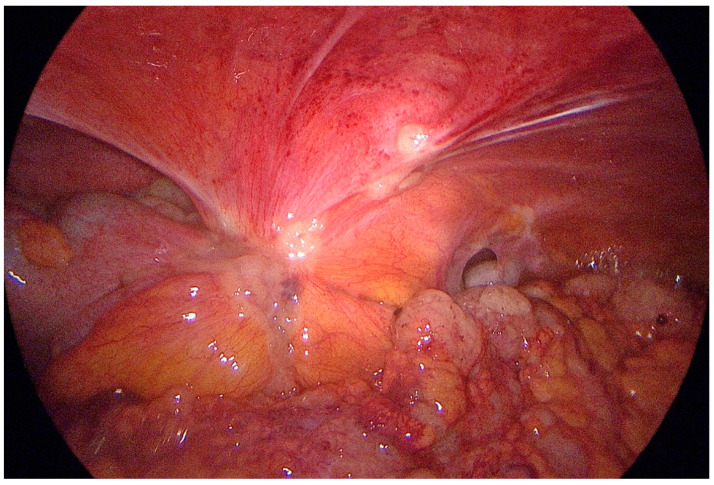
Peritoneal carcinomatosis of parietal peritoneum and sigmoid colon.

SL is performed under general anesthesia with the patient in a supine position. To begin the procedure, open Hasson, or optical ports-based technique, as well as Veress needle insufflation, can be used according to the surgeon’s personal experience. After the carbon dioxide pneumoperitoneum induction, the inspection of the peritoneal cavity through a laparoscope can start, looking for any signs of advanced or metastatic GC disease. Ascitic fluid is collected for cytology if present. The liver, spleen, diaphragm, mesentery, and peritoneal surfaces are examined for the presence of metastatic disease (Figure 1, Figure 2 and Figure 3). In case of no sign of distant GC extension, the left lateral lobe of the liver is elevated to expose the entire stomach. The gastric tumor is then carefully examined for any invasion beyond the serosal layer or infiltration of the nearby anatomical structures. If the tumor involves the posterior gastric wall, the exploration of the lesser sac may be needed to improve SL accuracy.

Although the SL technique has been long described in the literature, a standardized “Four-Step Procedure” has not been proposed until 2018 by Liu et al. [30].

The first step regards the exploration of the anterior abdominal wall and the surface of the visceral peritoneum. Specifically, the exploration of the anterior abdominal wall is performed with a circular clockwise orientation, starting from the diaphragmatic domes, the teres, and falciform ligaments, then proceeding towards the left side, the hypogastric, and the right side of the anterior abdominal wall. Conversely, the exploration of the surface of the abdominal viscera is performed according to an “S-shaped” sequence, starting from the anterosuperior surface of the left and right liver, proceeding to the greater omentum and the transverse colon, then to the left colon and the left paracolic gutter, finally ending with the surface of the small intestine, the right colon, and the right paracolic gutter.

The second step aims to explore the pelvic parietal and visceral peritoneum. This step takes advantage of a 30° Trendelenburg position and requires the placement of two 5 mm trocars in order to take the intestine out of the pelvis. At this point, the bilateral iliac fossa (and the annexes for female patients) are exposed, and the pelvic floor and the peritoneal reflection can be examined after lifting the bladder fundus (or uterus in females) and pulling the sigmoid colon.

The third step concerns the inspection of the small intestine and mesentery. The 30° Reverse Trendelenburg or supine position is required. The greater omentum and the transverse colon are lifted up, and the transverse mesocolon, the Treitz ligament, and the duodenojejunal junction are examined. Subsequently, the whole surface of the intestinal mesentery, together with the surface of the small intestine, are explored from the Treitz region to the ileocecal valve.

The fourth step regards the exploration of the stomach and the adjacent area. In the supine position, the greater omentum is replaced in its anatomical position to expose the anterior gastric surface and the greater curvature. Next, the stomach is lifted in order to evaluate the presence of posterior neoplastic infiltration. Then, the exploration of the lesser curvature and lesser omentum may require the lifting of the left liver. In the case of proximal GC, the distance between the tumor and cardia should be evaluated, while pylorus and duodenal bulb infiltration should be examined in the case of distal GC.

Finally, after the exploration of the hepatorenal recess, the opening of the gastrocolic ligament and the inspection of the lesser sac should be considered for posterior GC.

During the procedure, biopsies are performed when peritoneal dissemination is detected.

PC represents a useful diagnostic tool for detecting GC spreading to the peritoneal cavity. In order to obtain samples for cytologic analysis, a peritoneal lavage is performed through the infusion of 150 mL of saline solution into hepatorenal and splenic recesses during the first step, and the infusion of 200 mL into paracolic gutters and the pelvis during the second step.

Being a surgical procedure in every aspect, SL can be burdened by the same complications of other surgeries. Except for the anesthesiologic complications that may occur due to comorbidities and/or intolerance of the pneumoperitoneum, the perioperative course can still be complicated by inadvertent bowel perforation during manipulation, damage to other intra-abdominal structures, hemorrhage (especially after biopsy), infection, and incisional hernia. Nevertheless, the incidence of these complications is quite rare, and SL can be considered a safe procedure.

## 4. Performance and Clinical Significance of SL with PC

SL can detect unknown metastatic diseases in up to three out of five GC patients despite negative staging imaging [4,31,32,33,34,35]. The accuracy of SL for GC varies according to patients’ characteristics, surgeon’s experience, and disease extent. In 2006, Sarela et al. analyzed the efficacy of SL in 657 patients with potentially resectable GC over a 10-year period, highlighting a 31% rate of metastatic disease [36]. A meta-analysis of five studies with a total of 240 patients showed an overall sensitivity of 84.6% (95% CI: 0.747, 0.918) and an overall specificity of 100% (95% CI: 0.977, 1.00) of SL for detecting GC peritoneal metastasis [11]. Similarly, a systematic review by Leake et al. showed an overall accuracy, sensitivity, and specificity of SL for M staging ranging from 85 to 98.9%, 64.3 to 94%, and 80 to 100%, respectively [37]. Furthermore, SL changed the therapeutic indication and treatment in 8.5–59.6% of GC patients, avoiding unnecessary laparotomies in 8.5–43.8% of cases. A study by Gertsen et al. [38] has recently evaluated the performance of SL in a multicenter cohort of 394 GC patients. The authors reported higher accuracy for advanced cT3–T4 and diffuse-type GC with an overall sensitivity of 82% (95% CI, 70–91%) and a specificity of 78% (95% CI, 73–83%). Nevertheless, its limited usefulness for the evaluation of parenchymal organ lesions, such as non-superficial liver metastases and lymph node dissemination, represents one of the main limitations of SL.

Nowadays, the importance of SL is also enhanced by the possibility of assessing the Peritoneal Carcinomatosis Index (PCI) [20]. PCI is a numerical scoring system specifically designed to assess the burden of peritoneal dissemination in patients with tumors with peritoneal metastasis, including advanced gastric cancer. It provides a detailed assessment of the severity and distribution of tumor spreading within the peritoneal cavity, playing an important role in both staging and treatment planning. PCI is calculated by dividing the abdominal cavity into 13 areas based on specific anatomical landmarks. Once the tumor deposits are identified, the surgeon assigns a score of 0 to 3 for each area, depending on the size and extent of macroscopic tumor involvement. Specifically, a score of 0 represents the absence of visible tumor implants, while a score of 1 indicates the presence of small tumor nodules measuring less than 5 mm. A score of 2 is assigned when macroscopical nodules ranging from 5 mm to 5 cm are identified, while a score of 3 indicates the presence of nodules larger than 5 cm or of extensive tumor confluence. The total PCI score, thus, can range from 0 to 39. It provides a comprehensive overview of the peritoneal dissemination burden, correlating directly with patient overall survival and prognosis [39,40]. Furthermore, PCI is employed as a valuable tool in treatment decision-making. Patients with higher PCI are considered for systemic chemotherapy and/or palliative measures in order to improve their quality of life and manage symptoms. On the other hand, patients with lower PCI may be candidates for aggressive surgery aiming to remove all the macroscopic peritoneal metastasis, followed by the administration of hyperthermic intraperitoneal chemotherapy (HIPEC), which has the goal of clearing the residual microscopic deposits.

Indeed, during recent decades, cytoreductive surgery followed by HIPEC [41,42,43], as well as pressurized intraperitoneal aerosol chemotherapy (PIPAC) [44,45,46], has emerged as novel therapeutic options for patients with advanced GC with PC [47,48]. Despite the PCI cut-off of 12 proposed by Glehen et al. in 2012 [49,50,51], the current literature recommends a PCI lower than 7 to achieve a complete cytoreduction [52,53]. Based on the available data, the NCCN Panel recommends HIPEC or laparoscopic HIPEC as a therapeutic alternative for carefully selected stage IV patients in the setting of ongoing clinical trials only [12].

PC can further improve the efficacy of SL for GC staging through the identification of occult carcinomatosis [54]. Indeed, positive PC is associated with an independent prognostic factor for poor prognosis and recurrence following curative gastric resection [55,56,57,58]. A systematic review of the accuracy of peritoneal washing in GC patients revealed a disease recurrence rate of 11.1–100% and 0–51% in the case of positive and negative PC, respectively [59]. On the other hand, staging laparoscopy with PC plays a pivotal role also in gastric cancer re-staging after neoadjuvant chemotherapy. In a study by Mezhir et al., the authors found positive cytology in 291 out of a total of 1241 patients with gastric cancer who underwent staging laparoscopy with peritoneal washing from January 1993 to April 2009. Of these, a total of 48 patients with positive cytology underwent re-staging laparoscopy after the administration of systemic chemotherapy. The authors found a significantly improved disease-specific survival (DSS) in patients with a cleared cytology-positive GC after chemotherapy, as compared to patients who had persistently positive cytology (2.5 years vs. 1.4 years, *p* = 0.0003) [56]. Nevertheless, the role of radical gastrectomy for GC with positive PC remains unclear, as positive PC upgrades the stage disease to level IV, even in the absence of macroscopic PC.

Staging laparoscopy has some limitations that must be considered. It is an invasive procedure that carries a risk of complications, such as bleeding, infection, and injury to surrounding organs, albeit lower than the morbidity and mortality associated with nontherapeutic laparotomies [60,61]. Moreover, the accuracy of SL is still related to the surgeon’s expertise, and false-negative results can also occur due to the presence of micrometastases or the inability to biopsy all suspicious lesions [11,24,62,63].

## 5. Will There Still Be Room for SL in the Era of Artificial Intelligence?

SL has become part of the standard preoperative assessment of GC staging during recent decades. However, the continuous technological progress of CT imaging in terms of accuracy and precision [64] and the rise of new technologies, namely artificial intelligence (AI) and radiomics [65], are questioning the indications of SL for patients with advanced GC. Nowadays, CT is widely recommended for preoperative and post-treatment GC staging, having an accuracy ranging from 77.8% to 93.5% for T staging [66,67,68] and from 50% to 70% for N staging [69]. FDG-PET has a lower accuracy rate in preoperative staging of gastric cancer, due to low FDG uptake in diffuse and mucinous tumor types, which are common in this disease [70,71]. Compared to CT-scan, FDG-PET has lower sensitivity but higher specificity in the detection of lymph node involvement (56% vs. 78%, 92% vs. 62%, respectively) [72]. Recently, Borggreve et al. [73] reported that, while rarely employed for the diagnosis of peritoneal metastasis, IRM demonstrated the best diagnostic accuracy, sensitivity, and specificity as compared with other imaging modalities. In particular, the authors found that accuracy, sensitivity, and specificity for the detection of peritoneal dissemination of DWI-IRM and PET-CT were 83%, 84%, and 82% and 80%, 84%, and 73%, respectively [73]. In recent decades, the employment of MRI for GC staging has aroused interest due to its ability to provide greater tissue contrast resolution associated with the absence of ionizing radiation [74,75,76]. Two systematic reviews and meta-analyses evaluated the advantages of MRI in the preoperative staging of GC, highlighting the accuracy of MRI for T and N stages [77,78]. In 2012, Seevaratnam et al. reported an MRI overall accuracy of 82.9% and 85.3% for T and N staging, respectively [77]. Thereafter, in 2015, the subgroup analysis by Huang et al., only considering T3 and T4 GC, showed a pooled sensitivity and specificity of MRI of 93% and 91%, respectively [78]. Nevertheless, a more recent systematic review published by Laghi et al. in 2017 analyzed the diagnostic accuracy of CT and MRI for peritoneal metastases in a cohort of 630 patients with gastrointestinal malignancies. MRI showed higher specificity (86% vs. 83%) and sensitivity (88% vs. 86%) for the detection of PC when compared to CT scans, despite not reaching statistical significance [79]. Although CT still represents the prevalent non-invasive method to detect peritoneal carcinomatosis, its sensitivity in the case of occult peritoneal metastasis is significantly reduced [10]. Indeed, the evidence of peritoneal metastasis during SL or surgery missed by CT represents a current challenge, especially considering that curative R0 surgery is not indicated for stage IV GC [12,13].

A prospective study by Li et al. [80] analyzed the reliability of a 4-point CT score system based on the radiological characteristics of suspected peritoneal metastases. Specifically, the authors prospectively validated the 4-point CT score through a region-to-region comparison of the detection of occult peritoneal metastases between SL and preoperative CT in a cohort of 385 patients (as regards free peritoneum: 0—no abnormal sign; 1 (mild)—slightly and homogeneously increased fat density; 2 (moderate)—heterogeneously increased density; and 3 (severe)—heterogeneously and obviously increased density, multiple strands, curl sign, or blurred-margined small nodules; as regards visceral peritoneum: 0—no lines displayed; 1—slightly thickened line; 2—obviously thickened line with enhancement; and 3—obviously thickened line with enhancement and tiny nodules or a small number of ascites). This scoring system has shown a specificity of 76.45 and a sensitivity of 87.5% for the diagnosis of occult peritoneal metastases. Furthermore, only a 2% false-negative rate was associated with a CT score lower than 2, probably suggesting that SL might be avoided for patients with a score below the aforementioned threshold.

More recently, AI has been broadly investigated as a complementary tool due to its potential to overcome many limitations of SL and computed tomography for GC in terms of accuracy and safety [81,82]. The prediction of T stage [83,84,85,86,87,88] and lymph node status [86,87,88,89,90,91,92,93,94] in GC patients through AI-based models have been deeply investigated in the literature. In addition, AI can be employed in assisting decision making by predicting the risk of peritoneal metastasis and identifying the optimal treatment strategy based on patient-specific characteristics.

Dong et al. [95] improved a radiomic nomogram, based on the association of Lauren type with 266 imaging phenotypes of the primary GC and the adjacent peritoneum, for the identification of occult peritoneal metastasis revealing an area under curve of 0.920 (95% CI 0.862–0.978) in two external validation cohorts. Huang et al. developed a convolutional neural network model able to detect occult peritoneal metastasis in 544 patients, showing a sensitivity of 81%, a specificity of 87.5%, and an area under curve of 0.90 [96].

Recently, Jiang et al. [97] analyzed the use of a deep learning model to predict occult peritoneal metastases, based on preoperative computed tomography images, in a cohort of 1978 patients. The authors implemented a prediction model based on a densely connected convolutional network combined with long-short connections, able to process a 10-cm^2^ CT image of the primary tumor to estimate the probability of occult peritoneal metastasis. The Peritoneal Metastasis Network model achieved an area under the curve of 0.92 with a sensitivity of 87.5% and a specificity of 98.2% in the external validation cohort. Moreover, this non-invasive model was an independent predictor of occult peritoneal metastases at the multivariable analysis, with better performance when compared to the conventional clinicopathological factors.

Interestingly, Schnelldorfer et al. [62] tried to identify some optical features differentiating benign from malignant peritoneal lesions of gastrointestinal origin. Two images of each peritoneal lesion, which was biopsied during SL, were analyzed through an expert survey of 10 surgeons, the optical appearance of 3 experienced investigators, digital image processing, and machine learning. There was a 36 ± 19% rate of metastases misidentification in the expert survey and machine learning using a neural network, resulting in an unacceptable clinical efficacy (area under curve of only 0.47).

In conclusion, AI may have the potential to revolutionize the staging of GC and redesign the role of SL. Furthermore, the development of image-guided surgical tools might integrate the AI application. In fact, while they have already been investigated to guide lymphadenectomy during resective surgery [98], the use of tools such as fluorescence could also help to detect microscopic peritoneal metastasis, similar to what has already been reported for ovarian cancer [99]. However, the evidence in the current literature is still anecdotal, and AI has not yet been employed in routine clinical practice on large datasets. For these reasons, SL will likely continue to play a pivotal role in the management and preoperative assessment of GC in the near future.

## 6. Conclusions

SL is a key tool for preoperative GC staging, allowing an accurate assessment of disease extent and the detection of peritoneal metastases that may be missed by conventional preoperative imaging.

The high sensitivity and specificity of SL, together with its negligible rate of complications, earned it a top-notch spot in GC clinical decision making, thanks to the possibility of preventing unnecessary laparotomy in case of metastatic disease and identifying all patients with advanced disease who will not benefit from curative surgery.

The integration of new technologies into current clinical practice, such as AI-based models, has the potential to improve the accuracy of preoperative staging tools and might provide a more accurate patient selection for tailored treatment strategies.

## Figures and Tables

**Table 1:** Main cancer society recommendations on staging laparoscopy for gastric cancer.

Society	Recommendation
National Comprehensive Cancer Network (NCCN) [12]	Indicated in - Resectable T3 and/or N+ GC when preoperative therapy is not considered; - Resectable cT1b or higher GC, with or without indication for preoperative chemoradiation; - Medically fit patients with unresectable disease.
European Society for Medical Oncology (ESMO) [14]	Indicated in - Potentially resectable stage IB-III GC for patients who are also candidates for perioperative chemotherapy [III, B].
Society of American Gastrointestinal and Endoscopic Surgeons (SAGES) [15]	Indicated in - cT3-4 N0 M0 GC. Contraindicated in - Obstructive, hemorrhagic, or perforated GC needing palliative surgery; - Early-stage GC (T1-2); - History of previous abdominal surgery.
Japanese Gastric Cancer Association (JGCA) [16]	Weakly indicated in - Patients with advanced GC and high-risk predictors of peritoneal carcinomatosis (linitis plastic, Bormann type 3–4, poor histological differentiation, and suspected lymphadenopathy at CT scan).
Italian Research Group for Gastric Cancer (GIRCG) [18]	Recommended in cases deemed to be at risk of peritoneal carcinomatosis not visible or doubtful at CT examination. Required in many randomized clinical trials of adjuvant and neoadjuvant therapy. PC, although limited by low sensitivity, is a useful completion of the final pathological staging.

GC: gastric cancer and PC: peritoneal cytology.
